# Genomic reconstruction of 100 000-year grassland history in a forested country: population dynamics of specialist forbs

**DOI:** 10.1098/rsbl.2018.0577

**Published:** 2019-05-29

**Authors:** Yuichi Yamaura, Ayu Narita, Yoshinobu Kusumoto, Atsushi J. Nagano, Ayumi Tezuka, Toru Okamoto, Hikaru Takahara, Futoshi Nakamura, Yuji Isagi, David Lindenmayer

**Affiliations:** 1 Graduate School of Agriculture, Hokkaido University, Kita 9, Nishi 9, Kita-ku, Sapporo, Hokkaido 060-8589, Japan; 2 Department of Forest Vegetation, Forestry and Forest Products Research Institute, 1 Matunosato, Tsukuba, Ibaraki 305-8687, Japan; 3 Fenner School of Environment and Society, Australian National University, Canberra, ACT 2601, Australia; 4 Graduate School of Agriculture, Kyoto University, Kyoto 606-8502, Japan; 5 Forestry Research Institute, Hokkaido Research Organization, Bibai, Hokkaido 079-0198, Japan; 6 Institute for Agro-Environmental Sciences, National Agriculture and Food Research Organization, 3-1-3 Kannondai, Tsukuba, Ibaraki 305-8604, Japan; 7 Western Region Agricultural Research Center, National Agriculture and Food Research Organization, 2575 Senyu-cho, Zentsuji, Kagawa 765-0053, Japan; 8 Department of Plant Life Science, Faculty of Agriculture, Ryukoku University, Otsu, Shiga 520-2194, Japan; 9 Kansai Research Center, Forestry and Forest Products Research Institute, 68 Nagaikyutaroh, Momoyama, Fushimi, Kyoto, Kyoto 612-0855, Japan; 10 Graduate School of Life and Environmental Sciences, Kyoto Prefectural University, 1-5 Hangi, Shimogamo, Sakyo, Kyoto 606-8522, Japan

**Keywords:** Anthropocene, forestry, human disturbance, last glacial maximum, RADseq, site frequency spectrum

## Abstract

Grassland ecosystems worldwide have been extensively converted to other land uses and are globally imperiled. Because many grasslands have been maintained by human activities, understanding their origin and history is fundamentally important to better contemporary management. However, existing methods to reconstruct past vegetation can produce contrasting views on grassland history. Here, we inferred demographic histories of 40 populations of four grassland forb species throughout Japan using high-resolution genome sequences and model-flexible demographic simulation based on the site frequency spectrum. Although two species showed a slight decline in population size between 100 000–10 000 years ago, our results suggest that population sizes of studied species have been maintained within the range of 0.5–2.0 times the most recent estimates for at least 100 000 years across Japan. Our results suggest that greater than 90% declines in Japanese grasslands and subsequent losses of grassland species in the last 100 years are geologically and biologically important and will have substantial consequences for Japanese biota and culture. People have had critical roles in maintaining disturbance-dependent grassland ecosystems and biota in this warm and wet forested country. In these contexts, disturbances associated with forest harvesting and traditional extensive farming have the potential to maintain grassland ecosystems and can provide important opportunities to reconcile resource production and conservation of grassland biodiversity.

## 1. Introduction

Grasslands are imperiled ecosystems worldwide [1]. For example, globally since 1700, the conversion rate of natural grasslands to farmlands (50%) has been twice that of forests [2], and temperate grasslands are among the world's most poorly protected biomes [1]. Many grasslands have been maintained by human intervention and show abrupt declines. For example, in the past century, semi-natural grasslands (maintained by human intervention without a significant application of chemicals and sowing) in southeastern Sweden decreased by 96% via afforestation [3]. The origin and history of grasslands are therefore important for their contemporary management [4].

Japan has incurred greater than 90% declines in grasslands in the past 100 years [5]. Grasslands covered greater than 10% of the nation's land surface until 100 years ago and were maintained by people to produce forage for livestock, natural fertilizer and thatching for rooves [5]. The majority of Japan's modern and medieval grasslands were therefore semi-natural grasslands (called ‘grasslands’ unless otherwise stated). However, agricultural and social modernization resulted in grasslands being converted to plantations or being replaced by natural forests (due to the warm and wet climate, abandonment leads to grasslands succeeding into forests [6]). Now, 67% of Japan's land is forested [5], and grasslands have become one of the nation's most endangered ecosystems (although they have been maintained by humans), supporting many species of conservation concern [5].

But how important are current declines of grassland ecosystems and their biodiversity? How long have they been maintained in Japan? There are contrasting views about grassland ecosystems in Japan's geological history (table 1). After the Last Glacial Maximum (LGM), fossil pollen deposits indicate that dense forests replaced grasslands/open forests, which covered northern and highland areas [9–11]. Conversely, 17% of Japan's pedosphere is black soil [21], which is supposed to develop under the grasslands [7,22]. This suggests the long-term spatial spread of grasslands in Japan [8].

**Table 1.** Hypotheses about historical dynamics of open environments in Japan.

material/time	100 000–10 000 years ago	10 000–1000 years ago	1000–100 years ago	recent 100 years
black soil [7,8]	grasslands first developed by Palaeolithic people	grassland development was enhanced by Neolithic people	grassland development was further enhanced by medieval people	grasslands/open forests declined due to the diminished dependency on the natural resources by Japanese people
fossil pollen, phytolith, charcoal [9–16]	northern/high elevation areas were covered by grasslands/open forests with alpine grassland species due to the severe climate; grasslands developed in Kyushu island	fire frequently occurred 10 000 years ago, and grasslands/open forests developed; but after that, they were replaced by dense forests	grasslands/open forests appeared likely due to human disturbance	
land form [17,18]	open floodplain environment (wetland, grassland and gravel bar) was created by active periglacial sediment production on hill slope	open floodplain environment was expanded by active fluvial processes due to increased precipitation and sediment transport from upper stream	open floodplain environment was further expanded by increased sediment discharge via human forest use	open floodplain environment was reduced due to the limited flood disturbance by dam and levee construction
climate [19,20]	post last interglacial period: multiple rapid cooling/warming events occurred; LGM was included	post last glacial period: increased temperature and precipitation; stable climate	stable climate	Increasing temperature
this study	population size was increased (*S. japonica*)/maintained (*D. superbus*)/decreased (*P. scabiosifolia* and *S. officinalis*)	population size was maintained in four species	population size was maintained in four species	beyond inference (evolutionally too recent)

There is an alternative, integrative hypothesis proposing that grassland and its biodiversity, which flourished during the LGM partially due to the volcanic ash falls [8,23], were destined to decline with the increased temperature and humidity after the LGM. However, since then, Neolithic people had maintained grasslands to facilitate hunting by intentional burning. Grassland biodiversity was maintained as a result [7,8,12]. This anthropogenic refugia hypothesis highlights the intermixed roles of climate and people in maintaining grasslands and their associated biodiversity. Sakaguchi [8] investigated a valley deposit (up to 26 200 years before present) near Tokyo, central Japan and found the dominance of non-arboreal plants in pollens and spores (approx. 47% on average) as well as large amounts of charcoal. Based on the nation-wide spatial congruence of black soil and Neolithic remains (Jōmon period), Sakaguchi [8] suggested that Japanese prehistoric culture maintained grasslands for at least 26 000 years.

Notably, existing methods to reconstruct past vegetation have limitations. For example, pollen grains are preserved in anaerobic environments [24] and their analyses are typically applied to sediments from bogs and lakes [13]. Phytoliths and pollen grains cannot be accurately classified at the species level [14,15]. Black soil may form from volcanic ash soil [7,12] and its distribution may not indicate past grasslands. As an alternative approach, we inferred demographic dynamics of grassland plants at a national scale using high-resolution genome sequences and model-flexible simulation based on the site frequency spectrum (SFS). Based on Sakaguchi [8], we tested the prediction that populations of grasslands species have been maintained for the last 26 000 years throughout Japan, up until 100 years ago.

## 2. Material and methods

We focused our analysis on four flowering forb species (*Swertia japonica*, *Dianthus superbus*, *Patrinia scabiosifolia* and *Sanguisorba officinalis*), which are culturally important and used widely as cut flowers and in medicine in Japan (electronic supplementary material, table S1). They have different life-history traits, but their distributions are strictly confined to dry grasslands. We selected 25 remnant (semi-natural) grasslands throughout Japan (electronic supplementary material figures S1, S2; table S2) and sampled each species from 10 grasslands during 2013–2014. Within each grassland population, we collected leaves from individuals located several metres apart and used 11–13 individuals in the genetic analysis.

We extracted total genomic DNA from silica gel-dried leaf tissue from each individual. We developed a library for 100 base paired-end double digest restriction-site associated DNA sequencing (ddRAD-seq) [25] consisting of 120 individuals and sequenced in one lane of Illumina Hiseq2000 (Illumina, San Diego, CA, USA) by Macrogen for each of four species. Loci with single nucleotide polymorphisms (SNPs) were discovered and filtered by Stacks (ver. 1.44 [26]). We inferred demographic dynamics (temporal change in effective population size) of sampled populations from folded SNP frequency spectra using a recently developed model-flexible method by Stairway Plot v2 [27] (see electronic supplementary material, appendix S1 for details of the analysis). Individuals with less than 250 000 reads were treated as low-quality samples and not used in the demographic analysis (8–13 individuals per population were retained).

## 3. Results and discussion

**Figure 1.**
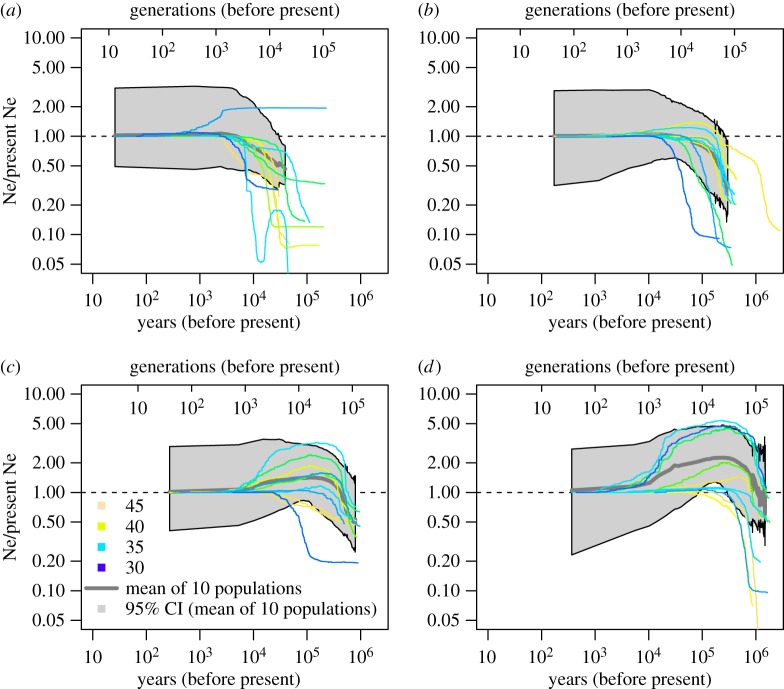
Estimates of effective population size for four species across Japan. Median estimates of 10 populations are shown with different colours by their latitude. Population size is scaled to the most recent value. Mean values of median estimates and 95% credible intervals across 10 populations are shown in grey shading. (*a*) *Swertia japonica*, (*b*) *Dianthus superbus*, (*c*) *Patrinia scabiosifolia* and (*d*) *Sanguisorba officinalis*.

We obtained greater than 120 × 10^6^ reads for each species from ddRAD-seq (electronic supplementary material, table S3). Demographic simulation inferred the population dynamics from 26 years ago (*S. japonica* [5–25 cm height]) to 2.8 Ma (*D. superbus* [30–50 cm]) (figure 1). Older dynamics of population size were estimated for a longer lifespan and taller height species (*P. scabiosifolia* [60–100 cm] and *S. officinalis* [30–100 cm]). In each species (except for *S. japonica*), effective population size was estimated to increase between 1000 and 100 thousand years ago (figure 1). Although stairway plots can yield spurious complex demographic patterns by over-interpreting the noise in the SFS (e.g. sequencing errors [28]), our inferred demographic histories, especially mean estimates, were rather simple. Demographic histories varied among populations but did not show clear latitudinal clines, suggesting the importance of grassland identity.

Our results from more recent periods were consistent with the prediction made at the outset of this investigation and suggested that historical population sizes of studied species were approximately within the range of 0.5–2.0 times the most recent estimates of population size for at least 100 000 years (figure 1). As discussed below, we suggest that grasslands and grassland species have likely been maintained in this predominantly forested country via interactions between climate, geomorphological processes and human activity. Agricultural and social modernization and constrained flood disturbance have reduced grassland ecosystems by greater than 90% in the past 100 years (table 1). This is biologically and geologically important and will have substantial consequences for Japanese biota and culture.

During 100–10 thousand years ago, *P. scabiosifolia* and *S. officinalis* decreased in population size while *D. superbus* maintained population size and *S. japonica* increased in population size (figure 1). *P. scabiosifolia* and *S. officinalis* may have decreased in population size due to the unstable climates during this generally cool period [19]. Nevertheless, we suggest that northern and highland grasslands/open forests would have been habitats for our study species since *P. scabiosifolia* and *S. officinalis* currently occur in boreal/polar regions including Siberia (electronic supplementary material, table S1). Human disturbance of natural vegetation and the resultant maintenance of semi-natural grasslands likely started from this period [7]. Two short lifespan and limited height species (*S. japonica* and *D. superbus*) may have been resistant to unstable severe climate and human disturbance via frequent dispersal and colonization of suitable habitats, especially the biennial *S. japonica* (*sensu* [29]).

From 10 000 years ago, no study species showed major changes in population size. Natural grasslands/open forests in northern and highland areas were replaced by dense forests during this period [9–11]. The anthropogenic refugia hypothesis states that grassland species would have shifted their major habitats to semi-natural grasslands [30], which were actively established/maintained after LGM [7]. Open floodplain environments expanded via active fluvial processes [17,18] and also would have become important grassland habitats [31]. In the southern and lowland areas, semi-natural grasslands may have persisted for tens of thousands of years [8].

Twentieth-century global environmental change suggests that the Earth is entering a new geological epoch—the ‘Anthropocene’ [20]. Conversely, Ellis *et al*. [32] suggested that ‘humans are ancestral shapers and stewards of Earth's terrestrial surface’. Semi-natural grasslands likely have been post-LGM refugia for grassland species in Japan [30,31]. Our results concur with these views and suggest that people have had critical roles in maintaining disturbance-dependent grassland ecosystems and biota in this warm and wet forested country. In these contexts, disturbances associated with forestry and traditional extensive farming have the potential to maintain grassland ecosystems and can provide important opportunities to reconcile resource production with conservation of grassland biodiversity [5,33].

## Supplementary Material

Appendix S1-2

## Data accessibility

Data are available from the Dryad Digital Repository at: https://doi.org/10.5061/dryad.f5032p5064 [34]. Other information supporting this article is uploaded as electronic supplementary material.
