# Supplementary material for: Genomic reconstruction of 100 000-year grassland history in a forested country: population dynamics of specialist forbs
Source: Biol Lett. 2019 May 29;15(5):20180577. doi: 10.1098/rsbl.2018.0577 (PMC6548723; doi:10.1098/rsbl.2018.0577)
Supplement: Appendix S1-2 [file rsbl20180577supp1.docx]

This appendix file describes sampling sites, species, and methods.

Appendix S1. Sampling sites and species.

Appendix S2. Full descriptions of the genetic analysis.

Manuscript title: Genomic reconstruction of 100,000-year grassland history in a forested country: population dynamics of specialist forbs

Authors: Yuichi Yamaura, Ayu Narita, Yoshinobu Kusumoto, Atsushi J. Nagano, Ayumi Tezuka, Toru Okamoto, Hikaru Takahara, Futoshi Nakamura, Yuji Isagi, David Lindenmayer

Appendix S1. Sampling sites and species.


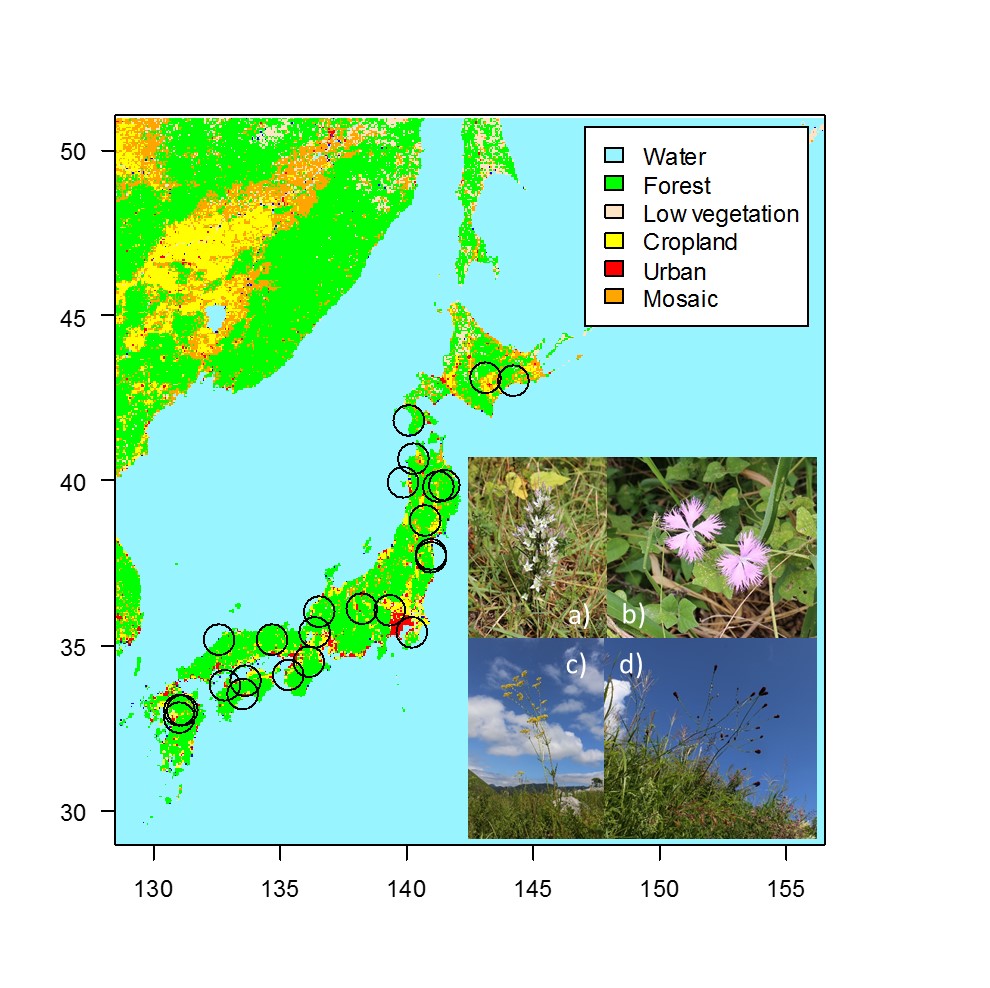


Fig. S1. Sampled grasslands and plant species.

Circles indicate the locations of individual grasslands, and 0.5-km resolution land-uses were from [1]. Four studied species are shown: (a) *Swertia japonica*, (b) *Dianthus superbus*, (c) *Patrinia scabiosifolia* and (d) *Sanguisorba officinalis*.


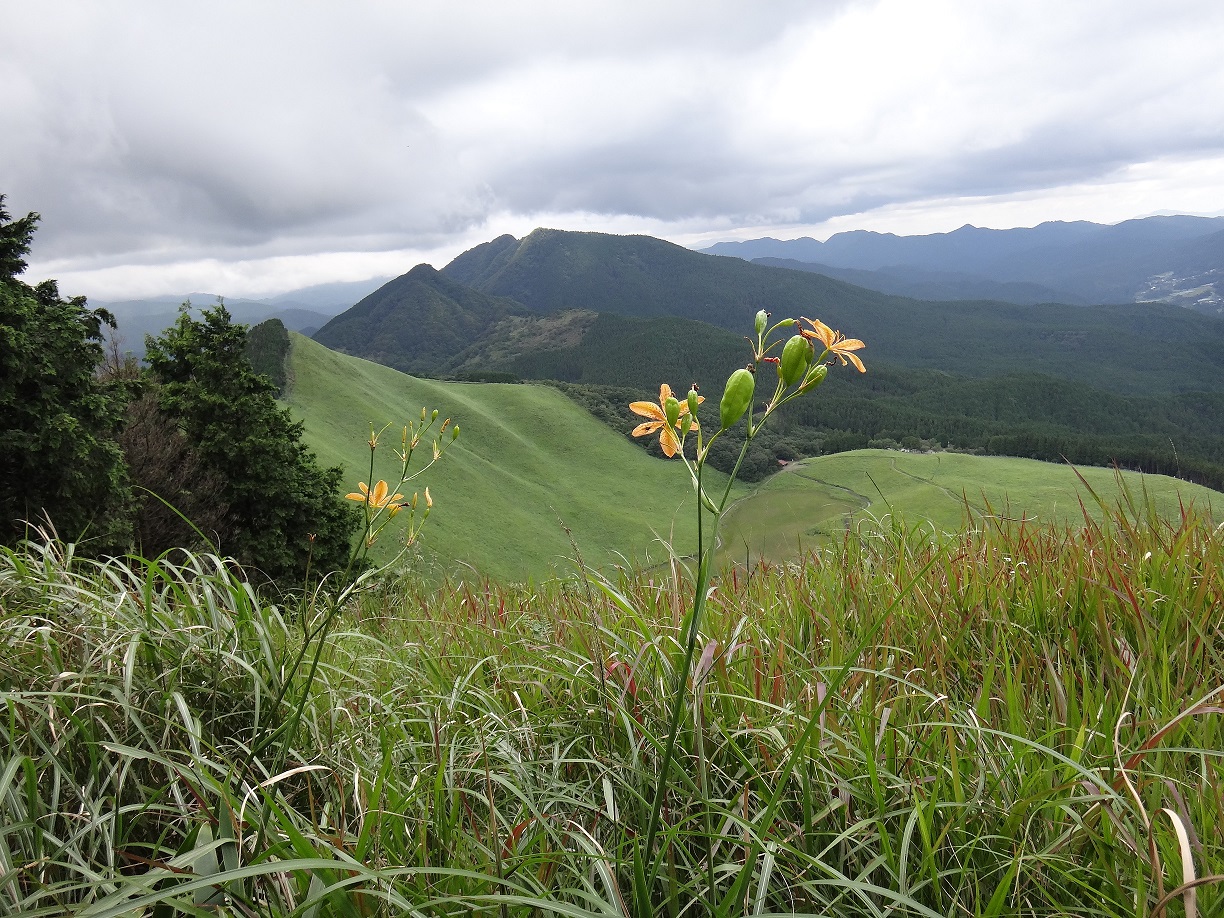


Fig. S2. A representative semi-natural grassland maintained by intentional burning (Soni plateau, Nara prefecture).

Remnant grassland is surrounded by natural and plantation forests.

Table S1. Life history traits of four studied species.

| Species | Distribution | Habitat | Plant height | Lifespan | Flowering period | Red list** |
| --- | --- | --- | --- | --- | --- | --- |
| *Swertia japonica* | Japan, Korea, China | Sunny, infertile grassland | 5-25 cm | 2 years  (2 years) | August to November | 13 |
| *Sw. tetrapetala** | Sakhalin, coastal Primorye, Hokkaido, Honshu | Same above | Same above | Same above | Same above | 2 |
| *Dianthus superbus* var*. longicalycinus* | Japan, Korea, China | Sunny grassland | 30-50 cm | Perennial  (4 years) | July to October | 10*** |
| *Patrinia scabiosifolia* | Kuril Islands, Sakhalin, Japan, China, eastern Siberia | Sunny grassland | 60-100 cm | Perennial  (7 years) | August to October | 19 |
| *Sanguisorba officinalis* | Japan, Korea, China, Europe, central Siberia | Sunny grassland | 30-100 cm | Perennial  (10 years) | August to October | 6 |
| *Sang. tenuifolia* var*. tenuifolia f. alba** | Kamchatkathe, Kuril Islands, Japan, Korea, China, eastern Siberia | Same above | Same above | Same above | Same above | 10 |

Note: Two species (*D. superbus* and *P. scabiosifolia*) are among the ‘seven autumnal flowers’, which have been of cultural significance for Japanese people for 1,200 years [2, 3]. All these species were formerly common in Japan until ~60 years ago but are now endangered in many prefectures. Although they have been culturally important and used as cut flowers and medicine, during the historical times, Japanese people would not have grown these species for particular purposes. Figures in the parentheses are the supposed lifespan used in the demographic simulation. *Since two species (*Sw. japonica* and *Sang. officinalis*) did not occur in the northern area, we instead sampled *Sw. tetrapetala* from one grassland and *Sang. tenuifolia var. tenuifolia f. alba* from three grasslands. **Number of Japanese prefectures (out of 47 prefectures), in which each species was registered in the prefectural red lists. Retrieved July 27th, 2017, from <http://jpnrdb.com/index.html>. ***Two sister species (*D.* superbus and *D. superbus var. longicalycinus*) are treated as one species.

Table S2. Sampled 25 grasslands in Japan.

| Site | Latitude | Longitude | Habitat | Size |
| --- | --- | --- | --- | --- |
| site1 | 33 | 131 | Grassland maintained by intentional burning | Large (>100 ha) |
| site2 | 33 | 131 | Grassland maintained by intentional burning | Large (>100 ha) |
| site3 | 33 | 131 | Grassland maintained by intentional burning | Large (>100 ha) |
| site4 | 34 | 134 | Grassland maintained by harvesting | Small (1-10 ha) |
| site5 | 34 | 133 | Pond bank | Tiny (0.1-1 ha) |
| site6 | 34 | 134 | Grassland maintained by intentional burning | Medium (10-100 ha) |
| site7 | 34 | 135 | Grassland maintained by intentional burning | Medium (10-100 ha) |
| site8 | 35 | 136 | Grassland maintained by intentional burning | Medium (10-100 ha) |
| site9 | 35 | 133 | Grassland maintained by intentional burning | Medium (10-100 ha) |
| site10 | 35 | 135 | Grassland maintained by intentional burning | Medium (10-100 ha) |
| site11 | 35 | 140 | Rice paddy bank | Tiny (0.1-1 ha) |
| site12 | 35 | 136 | Grassland maintained by harvesting | Small (1-10 ha) |
| site13 | 36 | 137 | Grassland maintained by harvesting | Tiny (0.1-1 ha) |
| site14 | 36 | 139 | Grassland maintained by harvesting | Tiny (0.1-1 ha) |
| site15 | 36 | 138 | Grassland maintained by intentional burning | Large (>100 ha) |
| site16 | 38 | 141 | Rice paddy bank | Tiny (0.1-1 ha) |
| site17 | 38 | 141 | Rice paddy bank | Tiny (0.1-1 ha) |
| site18 | 39 | 141 | Grassland maintained by harvesting | Small (1-10 ha) |
| site19 | 40 | 141 | Grassland maintained by intentional burning | Medium (10-100 ha) |
| site20 | 40 | 142 | Grassland maintained by harvesting | Tiny (0.1-1 ha) |
| site21 | 40 | 140 | Grassland maintained by intentional burning | Medium (10-100 ha) |
| site22 | 41 | 140 | Field bank | Small (1-10 ha) |
| site23 | 42 | 140 | Grassland maintained by harvesting | Small (1-10 ha) |
| site24 | 43 | 144 | Coastal grassland | Small (1-10 ha) |
| site25 | 43 | 143 | Road-side bank | Tiny (0.1-1 ha) |

Banks of agricultural fields or roads are periodically harvested by the owners. Management practices especially intentional burning are not always conducted annually. The selected grasslands varied in size (0.1 to 1,000 ha), management type (intentional burning and mowing) and location (e.g., hilltop areas, field banks).

Appendix S2. Full descriptions of the genetic analysis.

Total genomic DNA was extracted from silica gel dried leaf tissue of each individual using CTAB method [4], quantified with Qubit Fluorescent Assay and diluted in TE buffer to 5 ng/µL. A library for 100 base paired-end double digest Restriction Site Associated DNA sequencing (ddRAD-seq) [5] using the 6-base cutter *BglII* (A*GATCT) and *EcoRI* (G*AATTC), consisted of 120 individuals. The library was analyzed with a Bioanalyzer High Sensitivity DNA Kit. The average length of the library was 296 base pair (CV 18.6%) including 121 base of adaptor sequences. The library was sequenced in one lane of Illumina Hiseq2000 (Illumina, San Diego, CA, USA) by Macrogen for each of four species, which produced 127,204,147 (*Sanguisorba officinalis*)–249,958,446 (*Patrinia scabiosifolia*) reads (Table S3).

We removed the reads that contained low-quality base and adapter sequence from the raw sequence reads using Trimmomatic ver 0.33 [6] with following parameters: LEADING:19 TRAILING:19 SLIDINGWINDOW:30:20 AVGQUAL:20 MINLEN:100. Because Stacks (ver. 1.44 [7]) does not directly support paired-end reads, we used the data as 100 base single-end reads. Loci with single nucleotide polymorphisms (SNPs) were discovered and filtered by the pipeline *denovo_map.pl* in Stacks with following options: distance between stacks <3, number of mismatches allowed between tags =0. Minimum depth of coverage required for a stack was 3 and minimum stack depth for genotyping was 10.

The number of reads obtained for an individual ranged from 173,206–11,530,461 (*Swertia japonica*), 224,574–2,748,286 (*Dianthus superbus*), 25,553–6,715,471 (*Patrinia scabiosifolia*) and 31,965–3,359,572 (*Sanguisorba officinalis*) (Fig. S3). Individuals with less than 250,000 reads were treated as low-quality samples and not used in the following analysis (8–13 individuals per population were retained). Threshold of 250,000 reads was chosen to include at least two thirds of individuals for each population in the analysis. Loci showing excess heterozygosity (>0.5) were removed for possible homologous markers, and the loci genotyped for all individuals within each population were used in the analysis. The number of haplotypes were counted by TASSEL 5 [8] using Geno summary command.

Demographic dynamics (temporal changes in effective population size) of sampled populations were investigated from folded SNP site frequency spectra (SFS) using a recently developed model-flexible method (stairway plot) by Stairway Plot v2 [9]. The setting was modified as follows: 100bp × the number of polymorphic and monomorphic loci as length of sequence; folded SFS; random break point at 4, 8, 14 and 18 for populations with 9–13 individuals and 4, 8, 11, 15 for populations with 8 individuals; mutation rate = 7 × 10^-9^ [10]; lifespan (year per generation) was set to two for *Sw. japonica*, four for *Dianthus superbus*, seven for *P. scabiosifolia* and ten for *Sang. officinalis*.

Demographic history of a population was represented by median estimates of effective population size and associated 95% credible intervals (CIs) at the particular years. Absolute values of the most recent population size estimates greatly differed among the 10 populations within the same species (6-34 times). However, the absolute values of the estimates did not show major clines across the latitude. We therefore divided the median and 95% CI values by the most recent median estimate of each population for the comparison of temporal changes in population size within species. We then obtained mean values of median and CI values from ten populations within the same species. Since stairway plot provides more accurate prediction for recent demographic histories [9, 11], we obtained the mean values up to the most recent estimates even if there was only a single population estimate. The oldest years of species-level mean values varied among species depending on the number of populations that had older estimates: 40,000 (*Sw. japonica*)–1,500,000 years ago (*Sang. officinalis*). Since every population had different ‘bins’ of years in the trajectory of population size estimates yielded by the stairway plot, we obtained mean values for each of median and 95% CI values within every 800–5,000 years for each population (specific values differed among the species: Table S4). We finally obtained mean values of median and 95% CIs across ten populations.

Table S3. Numbers of reads, contigs and loci obtained from ddRAD-seq.

| Species | Total reads | Reads per individual | Total contigs |
| --- | --- | --- | --- |
| *Swertia japonica* | 215,236,357 | 1,585,430 | 1,095,155 |
| *Dianthus superbus* | 149,430,581 | 1,187,467 | 1,152,279 |
| *Patrinia scabiosifolia* | 249,958,446 | 1,920,363 | 1,149,430 |
| *Sanguisorba officinalis* | 127,204,147 | 968,513 | 711,749 |

For each species, 120 individuals were analyzed (480 individuals for four species).

Table S4. Parameters used to obtain mean values across populations.

|  |  | Population mean period* | |  | Inferred period** | |
| --- | --- | --- | --- | --- | --- | --- |
| Species | Bin size* | From | To |  | From | To |
| *Swertia japonica* | 800 | 26 | 40,000 |  | 26 | 227,565 |
| *Dianthus superbus* | 1,000 | 172 | 300,000 |  | 172 | 2,819,070 |
| *Patrinia scabiosifolia* | 5,000 | 272 | 800,000 |  | 272 | 973,382 |
| *Sanguisorba officinalis* | 5,000 | 366*** | 1,500,000 |  | 366*** | 1,806,126 |

*Break of years (‘bin’ size) and period (years before present) used to obtain mean values across 10 populations within species. **Range of inferred period (minimum and maximum values: years before present) by demographic simulation for ten populations within species. ***The population of ‘site 3’ yielded 1,599 succeeding same population size estimates at zero years ago. After these estimates, the calendar year jumped into 921 years ago with two succeeding size estimates, then 946 years ago with two succeeding size estimates, and so on. We considered that these most recent estimates at zero years ago were erroneous possibly because sequences were not sufficiently polymorphic, and we did not include them in the analysis. That is, they were not shown in Fig. 1 of the main text and not used to obtain population mean estimates.


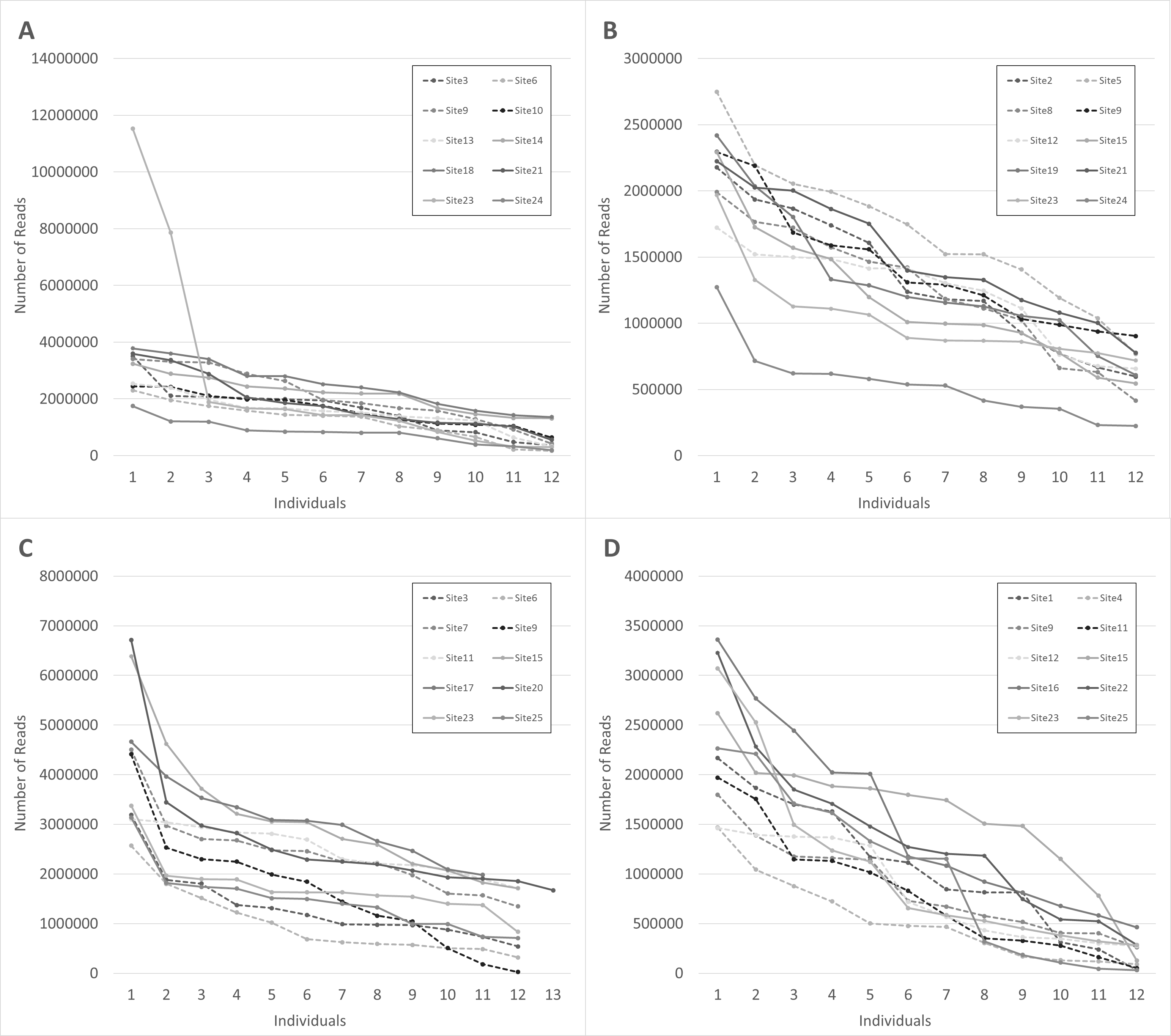


Fig. S3. Numbers of reads obtained for individuals in different populations. (A) *Swertia japonica*, (B) *Dianthus superbus*, (C) *Patrinia scabiosifolia* and (D) *Sanguisorba officinalis.* Individuals with less than 250,000 reads were excluded from the analysis.

Reference

[1] Broxton, P.D., Zeng, X., Sulla-Menashe, D. & Troch, P.A. 2014 A global land cover climatology using MODIS Data. *J. Appl. Meteor. Climatol.* **53**, 1593-1605. (doi:10.1175/JAMC-D-13-0270.1).

[2] Suka, T., Okamoto, T. & Ushimaru, A. 2012 *Grassland and Japanese: a journey of Japanese grassland for ten thousand years*. Tokyo, Tsukiji Shokan Publishing (in Japanese).

[3] Suka, T., Ushimaru, A. & Tanaka, Y. 2011 Grassland history, flora and insect fauna in the Japanese archipelago. In *Environmental history of grassland* (ed. T. Yumoto), pp. 101-122. Tokyo, Bun-ichi Sogo Shuppan (in Japanese).

[4] Milligan, B. 1992 Plant DNA isolation. In *Molecular genetic analysis of populations: a practical approach* (ed. A.R. Hoelzel), pp. 59-88. Oxford, IRL Press.

[5] Peterson, B.K., Weber, J.N., Kay, E.H., Fisher, H.S. & Hoekstra, H.E. 2012 Double digest RADseq: an inexpensive method for De Novo SNP discovery and genotyping in model and non-model species. *PLoS ONE* **7**, e37135. (doi:10.1371/journal.pone.0037135).

[6] Bolger, A.M., Lohse, M. & Usadel, B. 2014 Trimmomatic: a flexible trimmer for Illumina sequence data. *Bioinformatics* **30**, 2114-2120. (doi:10.1093/bioinformatics/btu170).

[7] Catchen, J., Hohenlohe, P.A., Bassham, S., Amores, A. & Cresko, W.A. 2013 Stacks: an analysis tool set for population genomics. *Mol. Ecol.* **22**, 3124-3140. (doi:10.1111/mec.12354).

[8] Bradbury, P.J., Zhang, Z., Kroon, D.E., Casstevens, T.M., Ramdoss, Y. & Buckler, E.S. 2007 TASSEL: software for association mapping of complex traits in diverse samples. *Bioinformatics* **23**, 2633-2635. (doi:10.1093/bioinformatics/btm308).

[9] Liu, X. & Fu, Y.-X. 2015 Exploring population size changes using SNP frequency spectra. *Nat. Genet.* **47**, 555. (doi:10.1038/ng.3254).

[10] Ossowski, S., Schneeberger, K., Lucas-Lledó, J.I., Warthmann, N., Clark, R.M., Shaw, R.G., Weigel, D. & Lynch, M. 2010 The rate and molecular spectrum of spontaneous mutations in *Arabidopsis thaliana*. *Science* **327**, 92-94. (doi:10.1126/science.1180677).

[11] Lapierre, M., Lambert, A. & Achaz, G. 2017 Accuracy of demographic inferences from the site frequency spectrum: the case of the Yoruba population. *Genetics* **206**, 439-449. (doi:10.1534/genetics.116.192708).
